# Scalar invariant transform based deep learning framework for detecting heart failures using ECG signals

**DOI:** 10.1038/s41598-024-53107-y

**Published:** 2024-02-01

**Authors:** Manas Ranjan Prusty, Trilok Nath Pandey, Pujala Shree Lekha, Gayatri Lellapalli, Annika Gupta

**Affiliations:** 1 Centre for Cyber Physical Systems, Vellore Institute of Technology, Chennai, 600127 Tamil Nadu India; 2 School of Computer Science and Engineering, Vellore Institute of Technology, Chennai, 600127 Tamil Nadu India; 3 School of Electrical Engineering, Vellore Institute of Technology, Chennai, 600127 Tamil Nadu India

**Keywords:** Computational biology and bioinformatics, Health care

## Abstract

Heart diseases are leading to death across the globe. Exact detection and treatment for heart disease in its early stages could potentially save lives. Electrocardiogram (ECG) is one of the tests that take measures of heartbeat fluctuations. The deviation in the signals from the normal sinus rhythm and different variations can help detect various heart conditions. This paper presents a novel approach to cardiac disease detection using an automated Convolutional Neural Network (CNN) system. Leveraging the Scale-Invariant Feature Transform (SIFT) for unique ECG signal image feature extraction, our model classifies signals into three categories: Arrhythmia (ARR), Congestive Heart Failure (CHF), and Normal Sinus Rhythm (NSR). The proposed model has been evaluated using 96 Arrhythmia, 30 CHF, and 36 NSR ECG signals, resulting in a total of 162 images for classification. Our proposed model achieved 99.78% accuracy and an F1 score of 99.78%, which is among one of the highest in the models which were recorded to date with this dataset. Along with the SIFT, we also used HOG and SURF techniques individually and applied the CNN model which achieved 99.45% and 78% accuracy respectively which proved that the SIFT–CNN model is a well-trained and performed model. Notably, our approach introduces significant novelty by combining SIFT with a custom CNN model, enhancing classification accuracy and offering a fresh perspective on cardiac arrhythmia detection. This SIFT–CNN model performed exceptionally well and better than all existing models which are used to classify heart diseases.

## Introduction

Cardiovascular diseases (CVD) are the diseases that affect the heart and the peripheral blood vessels^1^. CVDs are extremely deadly and life-threatening medical conditions, being most of the major reasons of global deaths. They have been a major health concern over the past 60 years^2^. These CVDs are not only complex; they are long term as well^3^.

Arrhythmia, or irregular heartbeat is one of the CVDs. It is a condition of the heart where the rhythm of the heart is abnormal under resting conditions. Arrhythmias occur when the electrical impulses to the heart that regulate the heartbeat do not function properly. Basically, electrical signals navigate from the top of the heart to the bottom, causing it to contract and pump blood. Therefore, the heart rate controls the speed and rhythm of the heart. However, the disruption of this system leads to irregular heartbeats that cause arrhythmias. If the heart does not pump blood effectively, important organs such as the brain, lungs, and other organs do not receive proper oxygenation, resulting in shutdown or damage^4^. Congestive Heart Failure (CHF) is another CVD that occurs when the heart is unable to pump blood normally. In this condition, the heart works less efficiently than normal. CHF is characterized by weakened heart muscle that contracts with less strength and efficiency^5^ and may be caused because of the heart muscles being too stiff, or the heart muscles being too weak to squeeze the blood out of the heart. Due to these reasons, blood moves slowly through the heart and body and as a result, rises the heart pressure. Thus, the heart is unable to carry enough nutrients and even oxygen to suffice the needs of the body^6^.

There are several ways of diagnosing CVDs. One of them is by analysing ECG signals. ECG is a simple diagnostic tool that is used to record the electrical fluctuation of heart. The ECG draws a record of the heart beats in the form of a graph. The electrical patterns in an ECG, representing each heartbeat, consist of distinctive peaks and valleys. This data serves dual purposes: evaluating the duration of heart electrical activity to determine its regularity and identifying the workload on different parts of the heart muscle. The ECG signal's frequency range is 0.05–100 Hz, with a dynamic range of 1–10 mV^7^.

A typical ECG signal is formed by its characteristic waves like P-wave, Q-wave, R- wave and S-wave (collectively known as the QRS complex) and T-wave in a certain order, duration and dimension. Sometimes, a wave called the U-wave follows the other five waves. It is usually of a smaller amplitude^8^. Successful ECG analysis hinges on precisely detecting the QRS complex, T-waves, and P-waves^9^.

Medically, the fluctuations in the specifications of ECG signals are looked at and interpreted manually, by the medical staff or doctors to detect any abnormalities and find specific heart conditions. But since ECG signals are not stationary, evidence of CVDs on the signal may be displayed arbitrarily on the timescale. Additionally, some important details are not recognizable manually and can lead to misinterpretations and errors^10^. It can also be very time-consuming and requires expertise in the domain. According to The American College of Cardiology Foundation, it takes 3500 supervised ECG reads to become an expert^11^. Therefore, this level of expertise is hard to attain, and also very time consuming. For these reasons, computer-aided techniques for ECG diagnosis may be more appropriate. The computerized methods help in overcoming the limitations which may be faced during manual assessment of ECG signals.

The motivations and contributions of the proposed work are discussed in Section "Related works". In Section "Motivation and contributions", we discuss the existing methods and related works. Section "Proposed approach" describes the approach used by us for detection of congestive heart failure, arrhythmia and normal sinus rhythm using Scalar Invariant Feature Transform based 2D-Deep CNN model in detail. Sections “Results and discussion” and “Conclusion” explain and discuss the results obtained and conclude the paper respectively.

## Related works

ECG interpretation is a skill that is necessary for most doctors^12,13^. Interpretation of an ECG and subsequently diagnosing based on it is complex and that requires combining the knowledge of many fields^14^. For a successful ECG diagnosis, one must know the rules of each diagnosis (i.e., the criteria for left ventricular hypertrophy), must correctly identify the ECG features (i.e., measure the R and S wave voltages), and must sort the relevant and irrelevant features of the ECG signal^15^. But it was found that physicians of all levels—right from medical students, residents, practicing physicians to cardiologists and cardiology fellows—had deficiencies in ECG interpretation^16^.

This led to the need to computerize the process of ECG interpretation and diagnosis. Presently, ECGs are often submitted with ECG-computer interpretation (ECG-CI), which are not always accurate. In this study by Chan et al.^17^, it was found that presenting ECG-CI along with the residents’ interpretation of the ECG improved their accuracy. This suggests that while ECG-CI by itself was not very accurate^18^, its presence increased the interpretation accuracy of the doctors. The following were some of the other models used to interpret and classify ECG signals. An Artificial Intelligence (AI) method was proposed by Lin^19^ that uses novel grey relational analysis (GRA) to classify ECG heartbeats. The MIT–BIH (Massachusetts Institute of Technology–Beth Israel Hospital) arrhythmia database was used for testing their method^20^ and it showed high accuracy for classifying ECG signals. In 2013, Luz et al.^21^ used the optimum-path forest (OPF) classifier for ECG heartbeat classification on the same MIT–BIH arrhythmia database. Mukhopadhyay and Krishnan^22^ proposed a method to process ECG signals that is saliency detection-based. Fast and accurate colour-based identification of different types of arrhythmias was made possible due to this model.

Then deep learning models of classifying ECG signals proved to be more effective and showed a higher accuracy compared to these preceding models. Deep learning is a part of machine learning (ML). It is enhanced by layers of neural networks, which are algorithms that roughly model how the human brain works. Training with large amounts of data composes neurons in neural networks. As a result, a deep learning model is created and new data is processed when training is complete^23^.

In 2016, Rahhal et al.^24^ came up with a deep neural network (DNN) model which used stacked denoising autoencoders (SDAEs) for feature learning, and later used a softmax regression layer on top giving the resultant DNN model. This approach was run on three databases—the MIT-BIH arrhythmia database^20^, INCART, and SVDB. Matthews et al.^25^ applied the Restricted Boltzmann Machine (RBM) and deep belief networks (DBN) to classify ECG. Not only that, their model could also detect ventricular and supraventricular heartbeats using single-lead ECG. Sannino and De Pietro^26^ presented a DNN composed of 7 hidden layers which was built empirically for the heartbeat classification. The effectiveness of both^25,26^ was tested using ECG signals from the MIT-BIH database^20^. Zhang et al.^27^ developed a model based on the deep learning approach to diagnose ECG automatically by employing the SHAPley Additive exPlanations (SHAP) method to enhance clinical interpretability.

The study^28^ introduces an innovative ensemble-based Support Vector Machine (SVM) classifier utilizing features like wavelets, high order statistics, R-R intervals, and morphological features, outperforming other classifiers with an impressive 94.4% accuracy on MIT-BIH arrhythmia database. Another study introduces a novel deep convolutional encoded feature (CEF) approach, employing a Bidirectional Long Short-Term Memory (BLSTM) network to detect arrhythmias from ECG signals. Comparative analysis against three other classifiers (ULSTM, GRU, and multilayer perceptron) is conducted on MIT-BIH arrhythmia database, classifying heartbeats into five categories. Results reveal the BLSTM network's exceptional performance, achieving a remarkable overall accuracy of 99.52% with a processing time of only 6.043 s^29^.

Several Convolutional Neural Network (CNN) models have been proposed over the years for classifying ECG signals. Nguyen et al.^30^ created a model that makes use of a segment based CNN model with Support Vector Machine (SVM) for recognition of atrial fibrillation without the need of using feature engineering. Their average F1 score came out as 84.19% under fivefold cross-validation. Yang et al.^31^ proposed a 12‑lead ECG arrhythmia classification method using a cascaded CNN and expert features. They also tested out the model for multiclass classification—9 categories—and obtained a final score of 86.5%. Porumb et al.^32^ proposed a model to detect CHF that makes use of raw ECG signals, rather than heart rate variability features. The accuracy for this model is prominent. The CHF data was taken from the BIDMC Congestive Heart Failure Database^33^.

Eltrass et al.^34^ proposed a model that combines CNN with Constant-Q Non-Stationary Gabor Transform (CQ-NSGT) to identify CHF and arrhythmia. Theirs is a multi-ECG diagnosis with an accuracy of 98.82%. Similarly, Çınar and Tuncer^35^ proposed a CNN model that identified CHF and Arrhythmia. The ECG signals were first classified by SVM and KNN algorithms, achieving 68.75% and 65.63% accuracy respectively. Then, they were classified with LSTM (Long Short Time Memory) yielding 90.67% accuracy. Lastly, Hybrid Alexnet-SVM algorithm was applied to the spectrograms of these signals and finally an accuracy of 96.77% was obtained^2^. Both the above methods used the MIT-BIH ARR database^20^, MIT-BIH Normal Sinus Rhythm (NSR)^36^, and BIDMC Congestive Heart Failure (CHF)^33^.

## Motivation and contributions

Customised CNN models, leveraging transfer learning architectures, demonstrate notable efficacy. These customized CNN models exhibit enhanced time complexity and accelerated learning rates, showcasing promising outcomes^37^. In this study, the SIFT method is used to extract feature signals converted images and a tailor-made multiclass CNN model has been implemented. This model will be called to as the SIFT–CNN model henceforth in this paper. The proposed model fundamentally takes ECG signals as image inputs and classifies them into three categories—arrhythmia, congestive heart failure and normal sinus rhythm—in the output. Particularly, ECG signals are used in this work because ECG machines are safe and inexpensive^38^. Hence these signals are easily available. The distinctiveness lies in the adoption of the Scale-Invariant Feature Transform (SIFT) for feature extraction from ECG signal images, offering a unique perspective compared to existing studies. Furthermore, the manuscript presents a custom-tailored Convolutional Neural Network (CNN) model, showcasing a novel experimental design that enhances the classification accuracy. The synergy between SIFT-based feature extraction and the custom CNN model constitutes a distinctive contribution, providing a fresh approach to cardiac arrhythmia detection. While we acknowledge certain limitations, such as the current lack of real-time accessibility, this manuscript significantly contributes to the theoretical framework by introducing a novel approach to feature extraction using the Scale-Invariant Feature Transform (SIFT). The empirical research aspect is strengthened through the integration of a custom Convolutional Neural Network (CNN) model, providing a unique and effective methodology for cardiac diseases detection. Practical applications are evident in the potential improvement of diagnostic accuracy, despite the acknowledged need for further development to enhance real-time accessibility, representing a positive step toward addressing these considerations in future research iterations.

The significant contributions of this study mentioned below.

1. The ECG signals have been denoised and normalized in order to decrease the value fluctuations.
2. Collected and processed signals were plotted and converted into images for further classification purpose.
3. SIFT, HOG and SURF methods are used for feature extraction process.
4. A novel SIFT based deep CNN architecture is been proposed for the classification of specified heart diseases.
5. Finally, the model has been validated and discussed.

## Proposed approach

**Figure 1:**
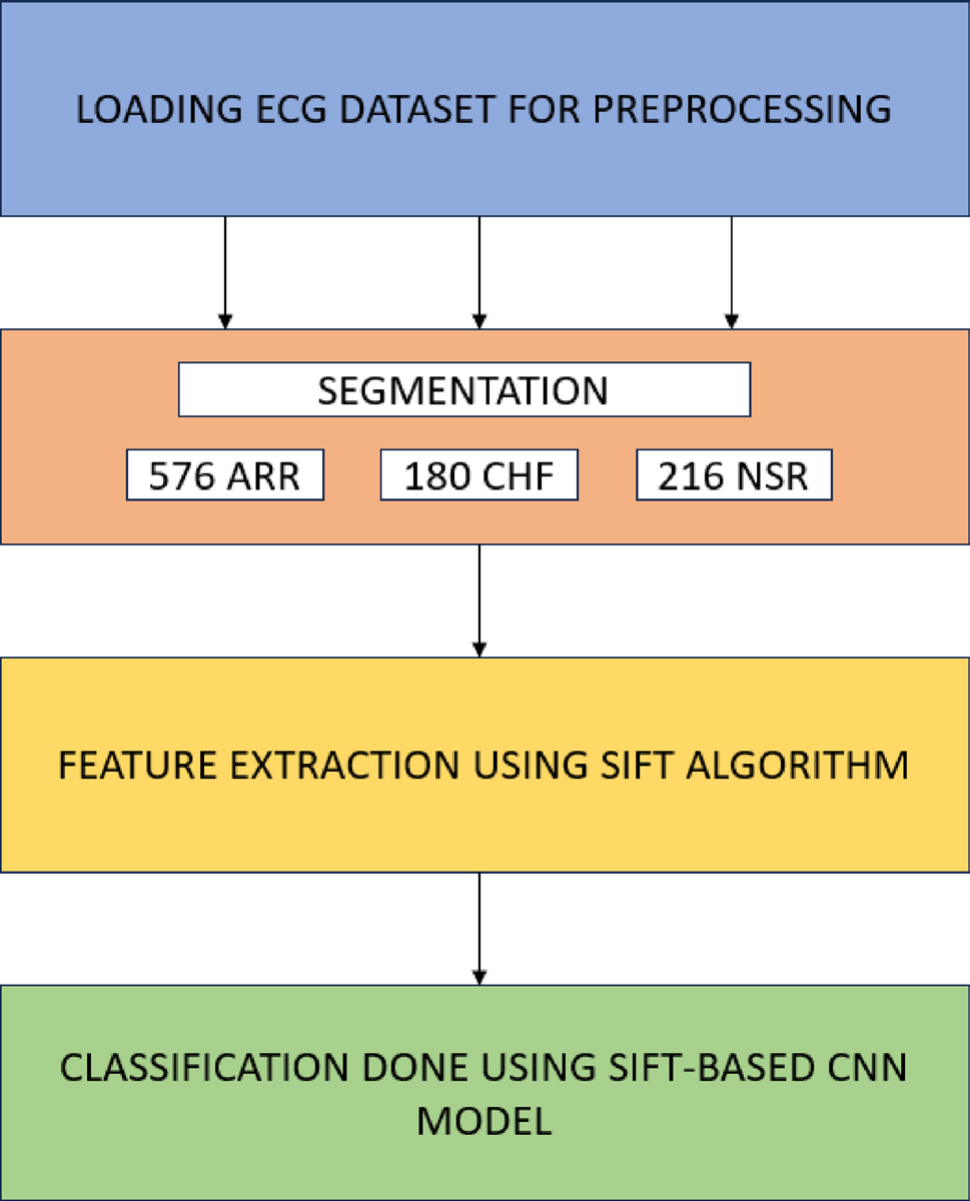
The structure of the proposed approach.

The proposed methodology contains mainly three stages. Firstly, in stage one the ECG signals are collected and a dataset is generated by pre-processing these signals with denoising and normalization. Secondly, these signals are transformed to images and the feature extraction is carried out using SIFT algorithm. Finally, the classification is carried out using the two-dimensional Deep CNN model. Figure 1 describes the proposed architecture of the SIFT–CNN model.

### ECG data collection and pre-processing

The SIFT–CNN model has been used to classify the selected signals into mainly three classes i.e. ARR, CHF, and NSR. A total of 162 signals were taken for analysis and classification from the following databases.

1. MIT-BIH ARR database—96 records^20^
2. MIT-BIH Normal Sinus Rhythm (NSR)—36 records^36^
3. BIDMC Congestive Heart Failure (CHF)—30 records^33^

**Figure 2:**
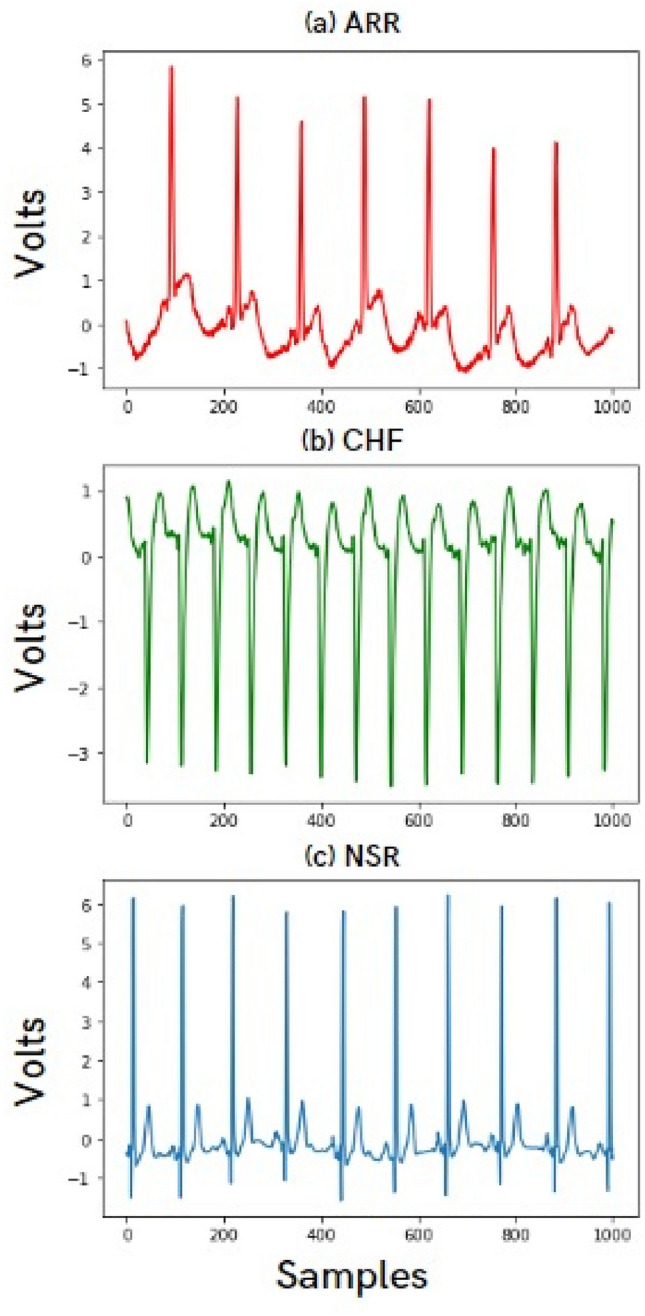
Sample ECG records of 1000 samples each for (**a**) ARR (**b**) CHF (**c**) NSR.

The final dataset had signals of three classes, namely Arrythmia, Congestive Heart Failure and Normal Sinus Rhythm. These ECG signals were in the form of tables, with each row as one ECG signal and each column as the y-axis value to be plotted to generate the signal. Each row in the table has been plotted to generate one ECG signal. Each ECG signal encompasses a few hundred beats. These signals are subsequently segmented into six distinct parts. Notably, the quantity of beats per segmented signal exhibits variability not only within each signal but also demonstrates differences across the three designated classes. There are a total of 65,535 columns, i.e., data points that are plotted for each signal. Each signal has further been divided with close to 10,000 data points for each segment. This resulted in a total of 972 signals that were plotted and stored as image files. These images have further been processed and have been run through the model. Figure 2 depicts a sample ECG record of 1000 samples for each of the three classes.

### Transformation of ECG signal to image and feature extraction using SIFT

A CNN model requires 2D images as inputs after the ECG data has been processed. SIFT algorithm is then used on this pre-processed data in order to extract important features from the image.

SIFT methodology was proposed by Lowe in 1999^39^ in order to solve the issue of scaling and rotation invariance in the feature extraction process. From the image, SIFT extracts a large collection of local feature vectors, each of which is resistant to changes in illumination, scale, translation, and geometry. SIFT characteristics are less impacted by noise and damaged pixels because of their spatial localization. A local feature vector called a SIFT descriptor is created by the SIFT algorithm from an image's data. The following three sections make up this algorithm:

*1. Detection of Scale Space Extrema*

The convolution interaction among capability and picture creates the scale-space picture $L(a,b,\sigma )$.The convolution takes place between the Gaussian Function^40^ $G\left(a,b,\sigma \right)$ and the image function $I(a.b)$. This is represented by Eq. (2) given below:

1 $$L\left(a, b, \sigma \right)=G(a,b,\sigma )\times I(a,b)$$

2 $$G\left(a,b,\sigma \right)= \frac{{e}^{\frac{-({a}^{2}+{b}^{2})}{2{\sigma }^{2}}}}{2\pi {\sigma }^{2}}$$

Equation (1) describes the creation of the scale-space image *L*(*a*,*b*,*σ*) through the convolution of the Gaussian function *G*(*a*,*b*,*σ*) with the image function *I*(*a*,*b*). This convolution represents the interaction between scale and image, forming a scale-space representation that is crucial for detecting features at different scales. Equation (2) defines the Gaussian function *G*(*a*,*b*,*σ*), which is a fundamental element in the convolution process. It represents a two-dimensional Gaussian distribution with spatial coordinates *a* and *b*, and the standard deviation *σ* controls the spread of the function. The Gaussian function plays a key role in smoothing and blurring, essential for feature detection in varying scales.

The significant parts of the picture called Difference of Gaussians (DoG) are removed by enhancing a measurable estimate of Gaussian's Laplacian. The DoG function is given by Eq. (3)

3 $$D\left(a, b, \sigma \right)=L(a,b,k\sigma )\times L(a,b,\sigma )$$

The DoG function is obtained by subtracting the blurred image at a larger scale *kσ* from the original image at scale *σ*. The DoG function highlights significant parts of the image, contributing to the identification of key points in scale-space. Here, $L(a,b,k\sigma )$ is the original image’s convolution $I(a.b)$ with the Gaussian blur $G(a,b,k\sigma )$ at scale $k\sigma$.

*2. Localization of Key points*

The primary points of the image are located at its extrema. It is necessary to reject the points over picture edges and those that are distinguished by poor contrast in order to choose the main point from image extrema when the major points are uncertain over image variation. When the Taylor expansion of scale-space function $D(a,b,\sigma )$ is done, it is shifted in such a way that the sample point becomes the origin. The equation is represented below:

4 $$D\left(a\right)=D+\left\{\left(\frac{\partial {D}^{T}}{\partial a}\right)\times a\right\}+\left\{\frac{{a}^{T}}{2}\times \left(\frac{{\partial }^{2}D}{\partial {a}^{2}}\right)\times a\right\}$$

The expression *D*(*a*) denotes the Taylor expansion of the scale-space function, a critical step in localizing key points and generating descriptors. In this context, *D* represents the value of the Difference of Gaussians (DoG) function at the sample point, capturing essential information about local image features. The terms $\frac{\partial {D}^{T}}{\partial a}$ and $\frac{{\partial }^{2}D}{\partial {a}^{2}}$ correspond to the first-order and second-order partial derivatives of *D* with respect to *a*, respectively. These derivatives play a crucial role in the Taylor expansion, providing information about the rate of change and curvature of the DoG function at the sampled point. Such computations are fundamental for achieving precise key point localization and generating robust descriptors in the SIFT algorithm, contributing to its effectiveness in scale-invariant feature extraction.

*3. Generation of Key Point Descriptor*

A local feature descriptor is then calculated for the area surrounding each key point.

Key point descriptors uses 16 histograms aligned in 4 × 4 grids, each with 8 orientation bins, making the feature vector of dimension 128^41^. This descriptor depends on a local picture gradient that has been turned by the direction of the central issue to offer direction invariance. A gradient orientation histogram is created nearby around the central issue to determine the descriptor direction.

Other feature extractions like HOG and SURF have also been tested on the images along with the SIFT algorithm. HOG descriptor centres on the construction and the shape of an image while SURF comprises of fixing a reproducible direction based on data from a round area around the key point. Then, at that point, a square district is developed and adjusted to the chosen direction and the SURF descriptor is extracted from it. Both these algorithms give an accuracy which is comparatively lesser than the accuracy given by SIFT, so, therefore a SIFT-based CNN model has been implemented.

There could be a few reasons why SIFT outperforms SURF for our model.

1. Dense Correspondences: SIFT provides denser correspondences, creating a more detailed set of matches between key points in images.
2. Performance and Computational Complexity: SURF is faster due to lower computational complexity, sacrificing some detail (correspondence density) for real-time processing advantages.
3. Descriptor Robustness: SIFT generates more distinctive and robust descriptors, contributing to more accurate and dense matches in specific scenarios.
4. Scale-Invariance: SIFT's scale-invariant nature allows it to perform well in situations with variations in scale.
5. Geometric Consistency: SIFT demonstrates better geometric consistency in matches, evident in the post-processing step to discard incompatible correspondences.^42^

### Classification with two-dimensional deep CNN

The dataset at this stage that consisted of a total of 972 images—576 ARR, 180 CHF and 216 NSR –underwent the Synthetic Minority Over-sampling Technique (SMOTE) to address class imbalance, thereby ensuring a balanced representation of data across all categories. This preprocessing step enhances the model's ability to generalize and effectively classify instances within each class, contributing to the overall robustness and reliability of the classification model^43^. In our classification, a deep CNN structure is employed and trained to train on extracted features of ECG signals. Though there are many existed architectures such as ResNet-50, VGG-16, ResNet-101, VGG-19, Inception-v3, AlexNET and DenseNet and we studied all of the above mentioned processes and examined their performance with SIFT–CNN architecture.

**Figure 3:**
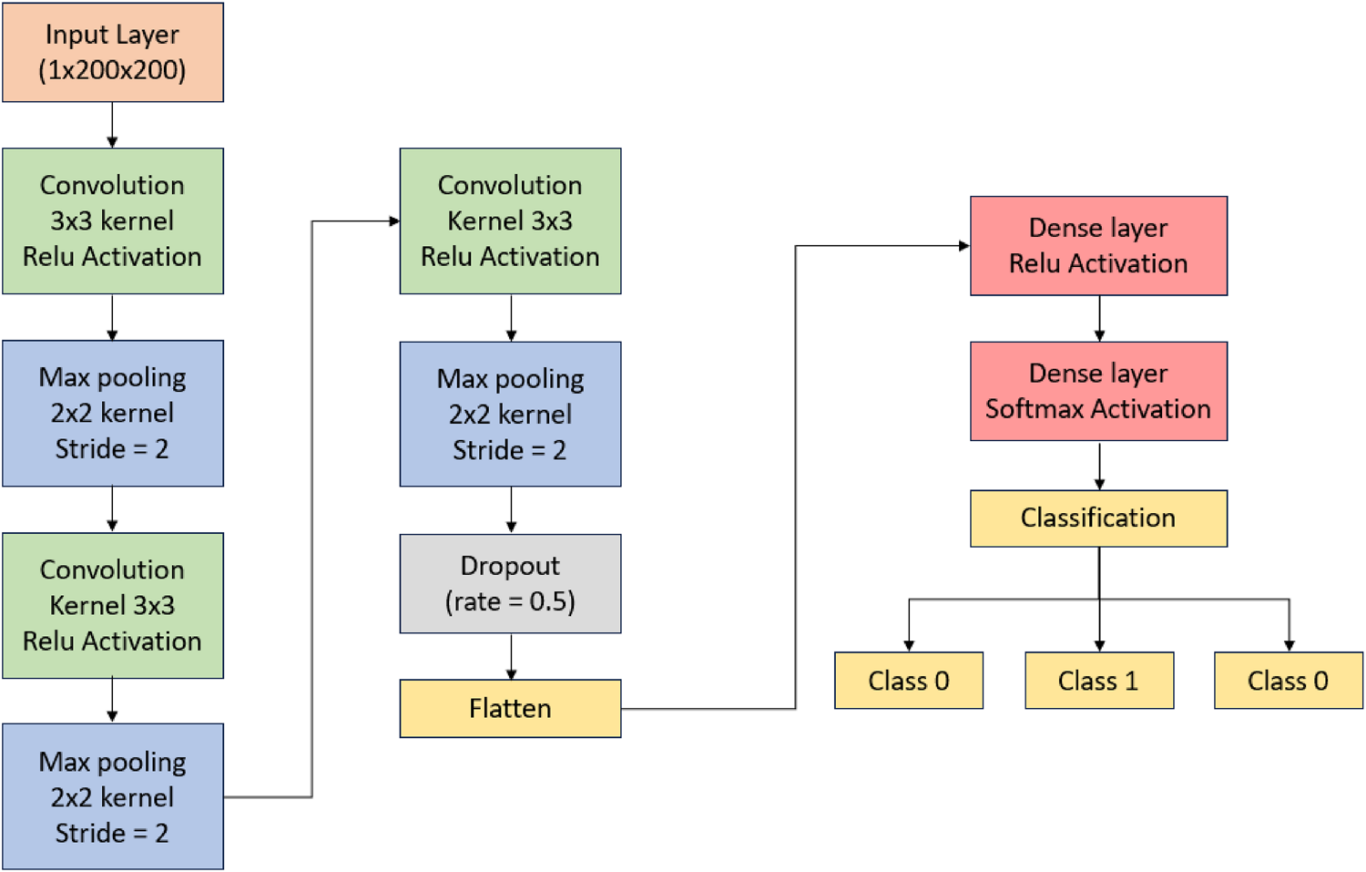
The proposed deep CNN architecture designed for heart disease classification.

SIFT–CNN model is trained on the extracted features in order to segregate the processed images into 3 different classes of heart diseases. SIFT–CNN model contains of 3 2-dimensional convolutional layers, 3 Max pooling layers and total 2 fully connected layers and in between the layers were drop out and flatten. Firstly, 1-dimensional ECG signal is converted to 2-dimensional RGB image. Then those images are properly balanced and fed to model as input as shown in Fig. 3. In the first layer, the RGB image in 200 × 200 × 3 dimension passes through a 32 of filter size 3 × 3 feature mapped convolutional layer with activation function as Rectified Linear Unit (ReLU). Then a 2 × 2 filter size of 2 stride max pooling layer is used to decrease the image dimension while keeping the prominent features of the ECG signal which is given as input secured. Then, dimension reduced images are fed to second convolutional layer which contains 32 of filter size 3 × 3 feature maps with activation function as ReLU. Again, a 2 × 2 filter size of 2 stride max pooling layer is applied on data. This process continued once again. By the end of third layer, all the input images will be dimensionally reduced with the important features kept intact. Then the output layers are flattened with drop out decimal 0.5. At last to provide final decision, the dense layer with soft-max as an output layer activation function that calculates the probability of result would be one of the possible classes.

In the comparison to other models, SIFT–CNN has many advantages. (i) By using fully connected layer followed by the dropout layer overfitting is reduced. (ii) Three Max—pooling layers were used for extract the features of signals carefully. (iii) Using “Relu” as an activation function, training process is speed up and the gradient explosion issue was solved.

Training the model is an iterative process. It can be trained by fixing various parameters like number of epochs, batch size and many more. One epoch is one complete execution of the training method over the entire loaded images. Since, decrease in the number of epochs cause under-fitting problems, selecting the number of epochs is an prominent task since increasing the number of epochs can lead to over-fitting. Here for the ECG dataset, optimal number of epochs are found at 30 epochs and batch size is taken as 16.

The CNN model serves as a crucial element in our methodology, particularly in the design of the SIFT–CNN model. The SIFT–CNN model's advantages include effective mitigation of overfitting, strategic use of Max-pooling layers, and efficient training through the application of the "ReLU" activation function. This model demonstrates robustness and advanced capabilities, leveraging CNN strengths for streamlined ECG signal classification.

Though CNN model don’t need feature extraction that aggregated from different pre-processing can be used to improve the network’s predictive ability. Therefore, SIFT–CNN model has been chosen. To predict the output, all the learnable features are merged with fully connected layer. To investigate the robustness of the model, further the model was validated using K-fold cross validation and also with other feature extraction methods. The chosen CNN architecture and hyperparameters were meticulously crafted to ensure optimal performance in ECG signal classification. By converting the 1-dimensional ECG signals into 2-dimensional RGB images, we effectively captured essential features for analysis. The CNN architecture, consisting of three convolutional layers with 32 filters, ReLU activation, and max-pooling, was designed to retain crucial signal features while minimizing overfitting. The incorporation of a fully connected layer, dropout mechanism, and three max-pooling layers demonstrated a strategic approach to enhance model robustness. The choice of ReLU activation expedited training, addressing potential gradient explosion issues. The selection of a batch size of 16 and 30 epochs, along with the Adam optimizer and categorical cross-entropy loss function, was based on a thoughtful iterative process. This approach considered the trade-off between under-fitting and over-fitting, with 30 epochs identified as the optimal balance. The chosen parameters aim to strike a harmonious balance, ensuring efficient training and robust performance on the ECG dataset. The hyper parameters of the proposed SIFT–CNN model for other techniques are given in Table 1.

**Table 1:** Hyper parameter table for the proposed SIFT–CNN for the classification of Heart.

Hyper parameters	Values for SIFT–CNN	Values for SURF–CNN	Values for HOG–CNN
ARR instances	576	576	576
CHF instances	576	576	576
NSR instances	576	576	576
Batch size	16	16	16
Epochs	30	10	25
Optimiser	Adam	Adam	Adam
Loss function	Categorical cross entropy	Categorical cross entropy	Categorical cross entropy

The standard technique for analysing the performance of a model is to find a confusion matrix and analysing it. This matrix finds the true positives (TP) i.e. number of instances that were classified into correct disease category, true negatives (TN) i.e. number of instances that were classified into normal heart beats, false positives (FP) i.e. number of instances that were classified into wrong category and false negatives (FN) i.e. number of instances that were classified into normal beats when they are actually not.^46^. To measure the performances of the models of we chose accuracy, precision, recall and F1-score as performance measures which are calculated from the confusion matrix. These metrics are calculated over 30 epochs with 5-folds cross-validation using the following formulae^46^.

5 $$Accuracy=\frac{TP+TN}{TP+TN+FN+FP}$$

6 $$Precision=\frac{TP}{TP+FP}$$

7 $$Recall=\frac{TP}{TP+FN}$$

8 $$F1 score=\frac{2 \times Precision\times Recall}{Precision+Recall}$$

9 $$Specificity = \frac{TN}{TN+FP}$$

In our study, we have classified the three types of heart diseases using CNN models with three different type of feature extraction techniques individually. The confusion matrix will describe how many ECG images are correctly classified into their respective class and vice versa. We have performed the classification using all three techniques and calculated the measures individually. Through the following analysis we decided that SIFT–CNN model is the best one.

## Results and discussions

**Figure 4:**
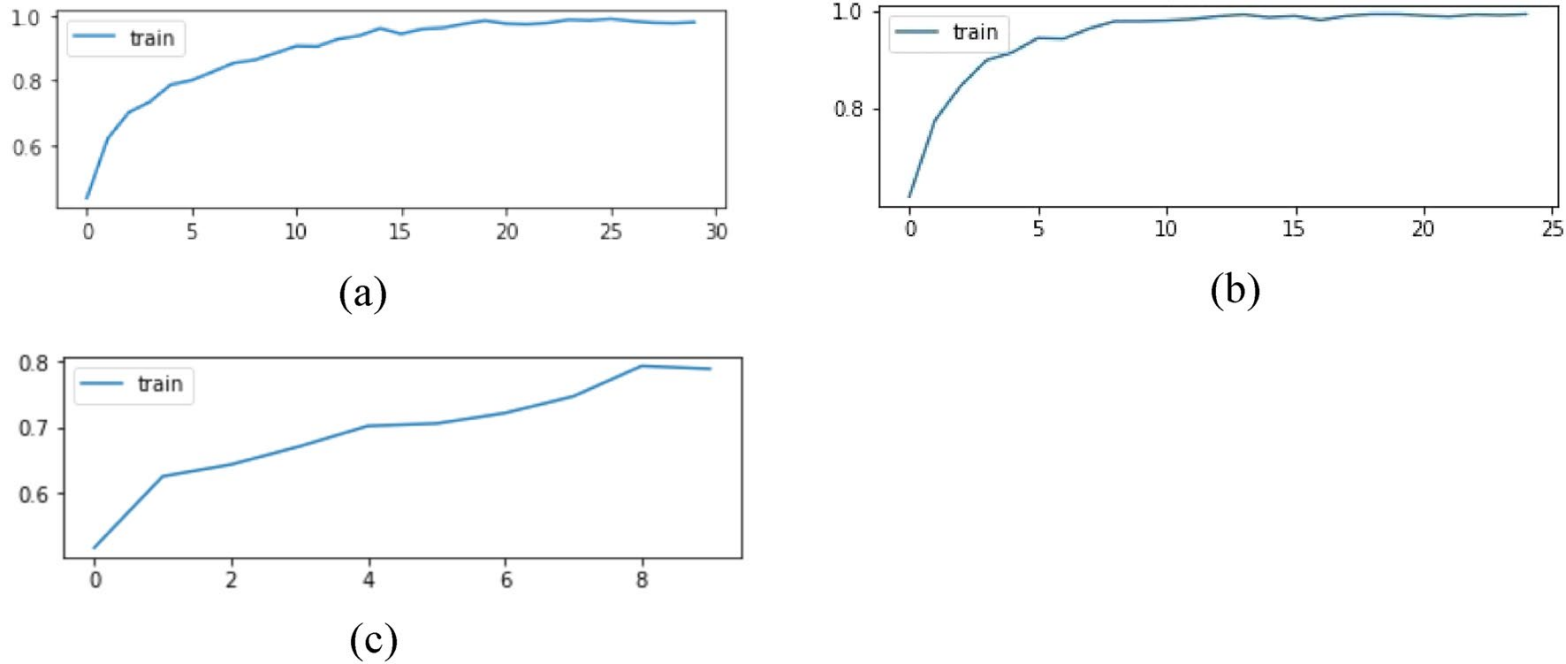
Accuracy vs epoch plots for accuracy for best performances for (**a**) graph of accuracy vs epochs for SIFT–CNN; (**b**) Graph of accuracy vs epochs for HOG–CNN; (**c**) graph of accuracy vs epochs for SURF–CNN.

In our study, after the denoising and normalisation, we have extracted the features from images SIFT algorithm and applied CNN model. Then again the features are extracted using SURF algorithm and suitable CNN model is applied and same process is repeated for HOG algorithm. For all these trials, the performance measures were calculated separately and compared with each other and then the best model is decided. In this section, we have presented the results obtained from the SIFT–CNN, HOG–CNN, SURF–CNN models in which the features are extracted through SIFT, HOG, SURF respectively. In Fig. 4, for all three graphs, the x-axis represents the number of epochs and the y-axis represents the accuracy.

**Figure 5:**
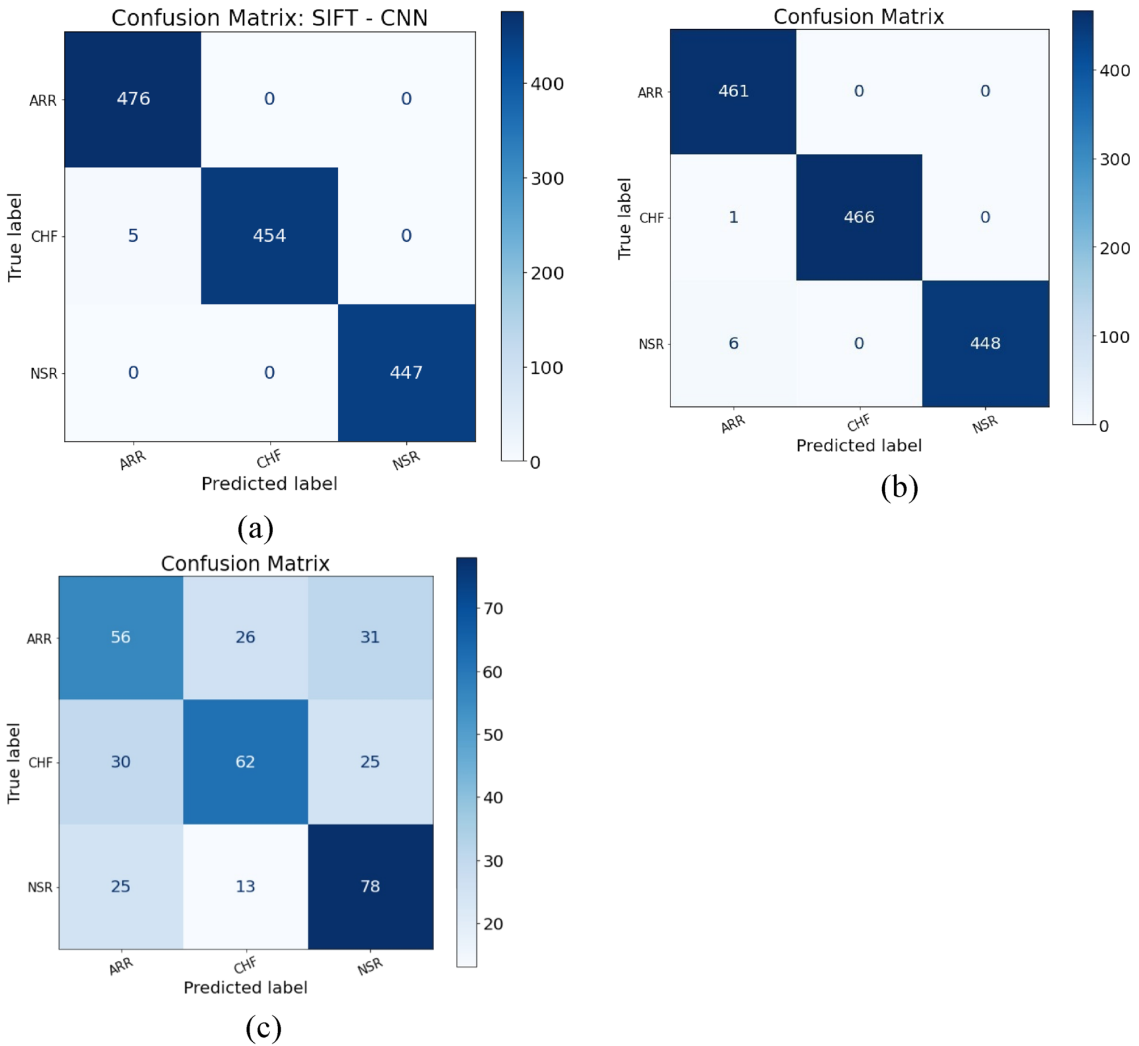
Confusion matrices of the best results constructed from the three models (**a**) Confusion matrix SIFT–CNN; (**b**) confusion matrix HOG–CNN; (**c**) confusion matrix SURF–CNN.

From the graph diagrams in Fig. 4, the accuracy for SIFT–CNN and HOG–CNN is constantly raising and pretty smoothly increasing whereas the accuracy of SURF–CNN model continuously fluctuating with each epoch and is not stable. The accuracy of SIFT–CNN and HOG–CNN reached nearly 1 at the end of epochs while SURF–CNN accuracy hardly reached 80%. Hence, in terms of accuracy and stability, SIFT–CNN is best model followed by HOG–CNN model. Figure 5 shows the confusion matrices for the best results from the three models. Table 2 shows the records of performance measures of each model along with the cross -validation measures. The SIFT–CNN model is processing 99.78% accuracy and while fivefold cross validation, the average accuracy reached to 99.92% which indicates that the model is well trained and can be accepted. Similarly, HOG–CNN accuracy reached 99.49% and the average accuracy reached to 99.96%. These two models can be accepted. But coming to the execution time, SIFT–CNN is taking nearly 18 min on average for 30 epochs but HOG–CNN is taking 22 min for 25 epochs. This is case with just 162 signals. The root mean square error (RMSE) value for SIFT-CNN, SURF-CNN and HOG-CNN was calculated to be 0.2375, 0.9722 and 0.2852 respectively for hold out cross validation. From this it is inferred that SIFT-CNN has the lowest RMSE hence it performs the best among the three. SURF-CNN performs the worst with the highest RMSE value.

**Table 2:** Diagnostic performance of SIFT–CNN model.

Modes	CNN multi-class classification of dataset (fivefold cross-validation)				CNN multi-class classification of dataset (Hold-out cross-validation)			
	ACC	PRE	REC	F1	ACC	PRE	REC	F1
SIFT	**0.9992**	**0.9991**	**0.9992**	**0.9991**	**0.9978**	**0.9978**	**0.9978**	**0.9978**
SURF	0.6915	0.6918	0.6918	0.6897	0.8350	0.8349	0.8349	0.8350
HOG	0.9996	0.9996	0.9996	0.9996	0.9949	0.9950	0.9949	0.9949

Significant values are in bold.

If we increase the dataset by adding more signals or increase epochs, the time will increase and ultimately, the difference between execution time of both models will be high. Hence, comparing both parameters, we can conclude that SIFT–CNN model is better than HOG–CNN. From the above information, SIFT–CNN model performed good in terms of accuracy, precision, recall and F1 score. Hence, it is confirmed that SIFT–CNN model is the best model among SURF–CNN and HOG–CNN model. Table 3 shows the comparison of all the models that already worked on classification on similar heart diseases. The models which have been worked on Arrhythmia, CHR and NSR were studied and all the metrics are recorded. These measures were all compared along with the proposed SIFT–CNN model in terms of accuracy and recall. Among all the models apart from the proposed model, the model with pre-trained CNN, Alex Net and CQ–NST transform performed good with 98.82% accuracy. Finally analysing and comparing all the existing models, we can conclude that the proposed SIFT–CNN model performs better than all existing in the classification of ARR, CHF AND NSR.

**Table 3:** Comparison of algorithms of the existing methods for the classification of Heart Diseases.

Ref. no	Method	Classification scheme	Accuracy	Recall/sensitivity	CV
^44^	K–NN	NSR vs CHF	87	80	Hold out
^45^	DT–SVM	NSR vs CHF	96.91	95.39	Hold out
^46^	LS–SVM	NSR vs CHF	98.21	98.07	Hold out
^47^	SVM	NSR vs CHF	90.95	91.31	Hold out
^48^	ApEn, DFA	NSR vs CHF	98.80	100	Hold out
^49^	CNN with CWT	NSR vs CHF vs ARR	93.75	93.06	Hold out
^50^	DCNN	NSR vs CHF	95.98	96.52	Hold out
^51^	DCNN with ensemble classifier	NSR vs CHF	87.54	76.71	Hold out
^34^	CNN, AlexNet and CQ-NSGT	NSR vs CHF vs ARR	98.82	98.87	Hold out
^52^	1D CNN	NSR vs ARR	96.3%	-	Hold out
^53^	AlexNet	NSR vs ARR	98.38%	-	Hold out
	(SURF–CNN)	ARR vs CHR vs NSR	83.50	83.49	Hold out
	(HOG–CNN)	ARR vs CHR vs NSR	99.49	99.49	Hold out
	**Proposed model (SIFT**–**CNN)**	**ARR vs CHR vs NSR**	**99.78%**	**99.78%**	**Hold out**
	**Proposed model (SIFT**–**CNN)**	**ARR vs CHR vs NSR**	**99.92%**	**99.91%**	**5**-**Fold**

Significant values are in bold.

**Figure 6:**
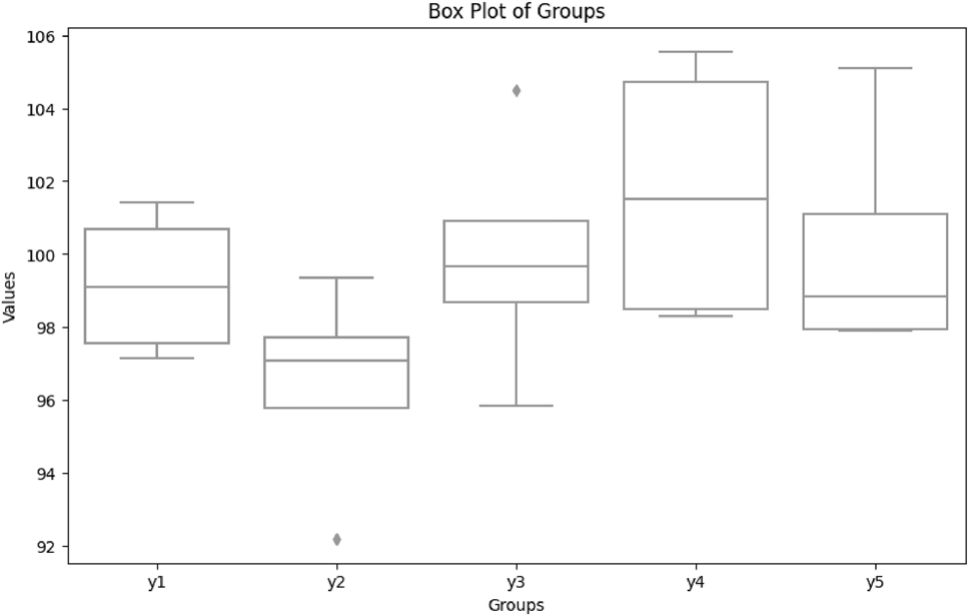
ANOVA test result graph.

**Figure 7:**
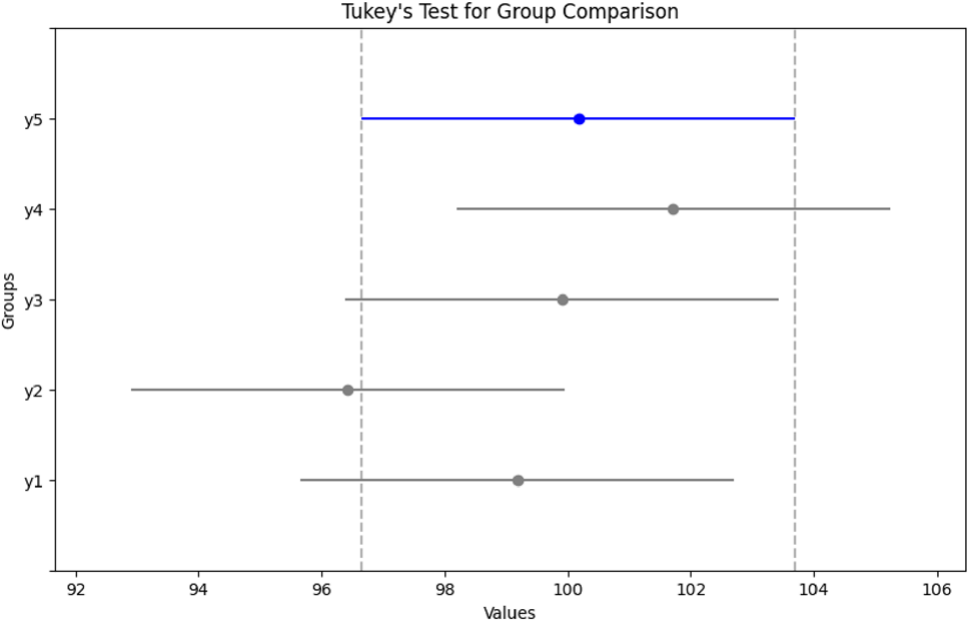
Tukey HSD test result graph.

The Analysis of Variance (ANOVA) test was employed to assess the statistical significance of the outcomes obtained from the proposed model, aiming to determine whether a notable difference exists in the performance when compared with other relevant models. The null hypothesis in ANOVA posits that there is no variance in means among the samples subjected to the test. In this test, the metrics of the top three performing models, as outlined in Table 4, along with out HOG-based CNN model were utilized to scrutinize the statistical significance of the proposed model in the classification task. Tukey's honestly significant difference test (Tukey's HSD) was applied to assess variations among sample means of the proposed model concerning other comparative models, gauging their significance. Figure 6 illustrates the ANOVA test result graph, while Fig. 7 displays the Tukey HSD test result graph, depicting the comparison between the proposed SIFT-CNN model and other models used for reference purposes.

**Table 4:** Summary of ANOVA test.

Source	SS	DF	MS	F	Prob > F
ANOVA table					
Columns	5.796	3	1.93184	1.45	0.2666
Error	210.778	16	13.17365		
Total	216.574	19

Table 4 displays the ANOVA results for the ECG classification task. The observed p-value (0.2666) surpasses the 0.05 threshold, indicating a lack of significant differences in the classification results among the compared models. This substantiates the acceptance of the null hypothesis (H0), affirming that the proposed model performs equivalently to the top three models in the comparison study. The Tukey HSD test corroborates this finding, revealing no significant disparity in the classification results between the proposed model and the other top-performing models subjected to the statistical test. Thus, the proposed model exhibits performance on par with these models, with the added advantage of enhanced computational efficiency.

The Matthews Correlation Coefficient (MCC) is a metric with a range from -1 to 1. Values close to 1 signify excellent predictions, indicating a strong positive correlation between predictions and true labels. This strong correlation implies that the predicted values closely align with the actual classifications. Conversely, when MCC is 0, there is no correlation between the variables, suggesting that the classifier is randomly assigning units to classes without any discernible link to their true values. In essence, MCC serves as a valuable metric for evaluating the performance of classification models, offering insights into the degree of agreement between predictions and actual class labels.

MCC can be calculated from a confusion matrix by using the formula:

10 $$MCC=\frac{\mathrm{c }\times {\text{s}}-{\sum }_{k}^{K}{p}_{k}\times {t}_{k}}{\sqrt{{(s}^{2}-{\sum }_{k}^{K}{{p}_{k}}^{2}){(s}^{2}-{\sum }_{k}^{K}{{t}_{k}}^{2})}}$$

To streamline the explanation, it is important to introduce the following intermediary parameters:

*c* = ${\sum }_{k}^{K}{C}_{kk}$, representing the overall count of correctly predicted elements.

s = ${\sum }_{i}^{K}{\sum }_{j}^{K}{C}_{ij}$, denoting the total count of elements in the confusion matrix.

${p}_{k}$= ${\sum }_{i}^{K}{C}_{ki},$ signifying the count of occurrences where class *k* was predicted (column total).

${t}_{k}$= ${\sum }_{i}^{K}{C}_{ik}$, indicating the count of occurrences where class *k* truly occurred (row total).

These intermediate variables facilitate a more concise representation of key metrics in the context of a confusion matrix^54^. The value of MCC for our model is 0.99479. This shows that the model gives excellent predictions.

Cohen's Kappa is a statistical measure that assesses the level of agreement between two raters or systems, corrected for chance agreement. It is commonly used in classification tasks, especially when dealing with categorical data or multiple classes.

11 $$K=\frac{{P}_{o}-{P}_{e}}{1-{P}_{e}}$$

where P_o_ represents the observed agreement, indicating the accuracy achieved by the model, while P_e_ stands for the expected accuracy, signifying the accuracy we would anticipate by chance alone^54^. If a model were to randomly assign units to classes while preserving the distribution of predicted classes, its accuracy should align with P_e_. For our model, the Cohen's Kappa is approximately 0.9444, indicating a high level of agreement beyond what would be expected by chance.

## Conclusion

In this paper, we have used the ECG dataset which consists of 96 ARR, 30 CHF and 36 NSR which then has been pre-processed and a dataset of 576 ARR, 180 CHF and 216 NSR was obtained. The model has been compiled by using SIFT feature extraction technique to extract important features from the image and a SIFT-based CNN model was developed for classification. The paper concludes that the SIFT algorithm produces the best result as compared to HOG and SURF algorithm by giving an accuracy of 99.2% in the fivefold cross validation and an accuracy of 99.7% in the hold out cross validation. Our model secured a high accuracy at 99.78%, standing out among other models, as evidenced by the comparison table. This notable achievement is coupled with optimal time efficiency, further highlighting its superiority over the alternatives. Advantages of the SIFT-CNN model include its exceptional accuracy, outperforming some other techniques in classifying ECG signals. However, a limitation lies in its real-time applicability, as the model's computational demands may hinder instantaneous processing in certain scenarios.

The future directions of our SIFT-CNN model could explore real-time applications in cardiac monitoring. Implementing a real-time framework would enable timely and continuous assessment of ECG signals, offering the potential for immediate detection and intervention in cardiac abnormalities. Additionally, extending the model to handle dynamic data streams and integrating with wearable devices could further enhance its practical utility in real-world, on-the-go healthcare scenarios. While recognizing this constraint, our model's future trajectory is aimed at transforming cardiac monitoring, fostering early intervention, and improving patient outcomes.

## Data Availability

The datasets generated during and/or analysed during the current study are available in the following databases. (1) MIT-BIH Arrhythmia Database, https://physionet.org/content/mitdb/1.0.0/. (2) The BIDMC Congestive Heart Failure Database, https://datamed.org/display-item.php?repository=0052&id=59028f725152c6571cffac71&query=. (3) The MIT-BIH Normal Sinus Rhythm Database, https://www.physionet.org/content/nsrdb/1.0.0/.

## References

[1] Wilhelmsen, L. Cardiovascular disease prevention. in *International Encyclopedia of Public Health (Second Edition)*, S. R. Quah, Ed., Oxford: Academic Press, pp. 438–447. doi: 10.1016/B978-0-12-803678-5.00055-2 (2017).

[2] Grimes DS (2012). An epidemic of coronary heart disease. QJM.

[3] Fuster V, Kelly B (2011). A conceptual strategy to address CVD and related chronic diseases in the developing world. Glob. Heart.

[4] “Arrhythmia—The Way your Heart Beats” , Narayana Health Care. Accessed: Jun. 26. [Online]. Available: https://www.narayanahealth.org/blog/arrhythmia-the-way-your-heart-beats/ (2022).

[5] Watson, E. L. Congestive heart failure. in *xPharm: The Comprehensive Pharmacology Reference*. S. J. Enna and D. B. Bylund, Eds., New York: Elsevier, pp. 1–6. 10.1016/B978-008055232-3.60613-0 (2007).

[6] Congestive Heart Failure and Heart Disease, WebMD. Accessed: Jun. 26, 2022. [Online]. Available: https://www.webmd.com/heart-disease/guide-heart-failure.

[7] Narayana KVL, Rao AB (2011). Wavelet based QRS detection in ECG using MATLAB. Innov. Syst. Des. Eng..

[8] Pal, S. ECG monitoring: Present status and future trend. In *Encyclopedia of Biomedical Engineering*, Elsevier, pp. 363–379. 10.1016/B978-0-12-801238-3.10892-X (2019).

[9] Anuradha B, Kumar K (2008). Classification of cardiac signals using time domain methods. ARPN J. Eng. Appl. Sci..

[10] Rajendra Acharya U, Kannathal N, Mei Hua L, Mei Yi L (2005). Study of heart rate variability signals at sitting and lying postures. J. Bodyw. Mov. Ther..

[11] Myerburg RJ, Chaitman BR, Ewy GA, Lauer MS (2008). Task force 2: Training in electrocardiography, ambulatory electrocardiography, and exercise testing. J. Am. Coll. Cardiol..

[12] Antiperovitch P (2018). Proposed In-training electrocardiogram interpretation competencies for undergraduate and postgraduate trainees. J. Hosp. Med..

[13] Salerno SM, Alguire PC, Waxman HS (2003). Training and competency evaluation for interpretation of 12-lead electrocardiograms: Recommendations from the American college of physicians*. Ann. Intern. Med..

[14] Wood G, Batt J, Appelboam A, Harris A, Wilson MR (2014). Exploring the impact of expertise, clinical history, and visual search on electrocardiogram interpretation. Med. Decis. Making.

[15] Hatala RM, Brooks LR, Norman GR (2003). Practice makes perfect: The critical role of mixed practice in the acquisition of ECG interpretation skills. Adv. Health Sci. Educ..

[16] Cook DA, Oh S-Y, Pusic MV (2020). Accuracy of physicians’ electrocardiogram interpretations: A systematic review and meta-analysis. JAMA Intern. Med..

[17] Chan AY, Mangat I, Casella L, Janevski J, Dorian P, Yu EH (2016). ECG computer interpretation and cardiology trainees: Help or hinderance?. Can. J. Cardiol..

[18] Padayachee C, Sear C, Challa P, Jenkins C, Whitman M (2018). Can the computer tell me what’s wrong with my heart? Early day lessons from digital hospital and ECG interpretation. Heart Lung Circ..

[19] Lin C-H (2008). Frequency-domain features for ECG beat discrimination using grey relational analysis-based classifier. Comput. Math. Appl..

[20] Moody, G. B., & Mark, R. G. MIT-BIH arrhythmia database. physionet.org. 10.13026/C2F305 (1992).

[21] Luz EJ, Nunes TM, de Albuquerque VHC, Papa JP, Menotti D (2013). ECG arrhythmia classification based on optimum-path forest. Expert Syst. Appl..

[22] Mukhopadhyay SK, Krishnan S (2022). Visual saliency detection approach for long-term ECG analysis. Comput. Methods Programs Biomed..

[23] “Do you know what deep learning is?” Accessed: Jun. 28, 2022. [Online]. Available: https://www.oracle.com/data-science/machine-learning/what-is-deep-learning/.

[24] Rahhal MMA, Bazi Y, AlHichri H, Alajlan N, Melgani F, Yager RR (2016). Deep learning approach for active classification of electrocardiogram signals. Inf. Sci..

[25] Mathews SM, Kambhamettu C, Barner KE (2018). A novel application of deep learning for single-lead ECG classification. Comput. Biol. Med..

[26] Sannino G, De Pietro G (2018). A deep learning approach for ECG-based heartbeat classification for arrhythmia detection. Future Gener. Comput. Syst..

[27] Zhang D, Yang S, Yuan X, Zhang P (2021). Interpretable deep learning for automatic diagnosis of 12-lead electrocardiogram. iScience.

[28] Pandey SK, Janghel RR, Vani V (2020). Patient specific machine learning models for ECG signal classification. Proc. Comput. Sci..

[29] Pandey SK, Janghel RR (2021). Automated detection of arrhythmia from electrocardiogram signal based on new convolutional encoded features with bidirectional long short-term memory network classifier. Phys. Eng. Sci. Med..

[30] Nguyen QH, Nguyen BP, Nguyen TB, Do TTT, Mbinta JF, Simpson CR (2021). Stacking segment-based CNN with SVM for recognition of atrial fibrillation from single-lead ECG recordings. Biomed. Signal Process. Control.

[31] Yang X, Zhang X, Yang M, Zhang L (2021). 12-Lead ECG arrhythmia classification using cascaded convolutional neural network and expert feature. J. Electrocardiol..

[32] Porumb M, Iadanza E, Massaro S, Pecchia L (2020). A convolutional neural network approach to detect congestive heart failure. Biomed. Signal Process. Control.

[33] Baim, D. S., *et al.* The BIDMC congestive heart failure database. physionet.org. 10.13026/C29G60 (2000).

[34] Eltrass AS, Tayel MB, Ammar AI (2021). A new automated CNN deep learning approach for identification of ECG congestive heart failure and arrhythmia using constant-Q non-stationary Gabor transform. Biomed. Signal Process. Control.

[35] Çınar A, Tuncer SA (2021). Classification of normal sinus rhythm, abnormal arrhythmia and congestive heart failure ECG signals using LSTM and hybrid CNN-SVM deep neural networks. Comput. Methods Biomech. Biomed. Engin..

[36] T. A. L. The Beth Israel Deaconess Medical Center. The MIT-BIH Normal Sinus Rhythm Database. physionet.org. 10.13026/C2NK5R (1990).

[37] Ribeiro AH (2020). Automatic diagnosis of the 12-lead ECG using a deep neural network. Nat. Commun..

[38] Aziz S, Ahmed S, Alouini M-S (2021). ECG-based machine-learning algorithms for heartbeat classification. Sci. Rep..

[39] Păvăloi I, Ignat A (2019). Iris image classification using SIFT features. Proc. Comput. Sci..

[40] Azeem A, Sharif M, Shah JH, Raza M (2015). Hexagonal scale invariant feature transform (H-SIFT) for facial feature extraction. J. Appl. Res. Technol..

[41] Shiji TP, Remya S, Thomas V (2017). Computer aided segmentation of breast ultrasound images using scale invariant feature transform (SIFT) and bag of features. Proc. Comput. Sci..

[42] Oyallon E, Rabin J (2015). An analysis of the SURF method. Image Process. Line.

[43] Chawla NV, Bowyer KW, Hall LO, Kegelmeyer WP (2002). SMOTE: Synthetic Minority Over-sampling Technique. J. Artif. Intell. Res..

[44] Cornforth, D. J., & Jelinek, H. F. Detection of congestive heart failure using Renyi entropy. In *2016 Computing in Cardiology Conference (CinC)*, pp. 669–672 (2016).

[45] Chen W, Zheng L, Li K, Wang Q, Liu G, Jiang Q (2016). A novel and effective method for congestive heart failure detection and quantification using dynamic heart rate variability measurement. PloS One.

[46] Kumar M, Pachori RB, Acharya UR (2017). Use of accumulated entropies for automated detection of congestive heart failure in flexible analytic wavelet transform framework based on short-term HRV signals. Entropy.

[47] Wang Y (2018). Comparison of time-domain, frequency-domain and non-linear analysis for distinguishing congestive heart failure patients from normal sinus rhythm subjects. Biomed. Signal Process. Control.

[48] Isler Y, Narin A, Ozer M, Perc M (2019). Multi-stage classification of congestive heart failure based on short-term heart rate variability. Chaos Solitons Fractals.

[49] Tabaa, M., Dellagi, S., Abbas, D., Moutaouakkil, F., & Karboub, K. Full training convolutional neural network for ECG signals classification. 10.1063/1.5138541 (2019).

[50] Acharya UR (2019). Deep convolutional neural network for the automated diagnosis of congestive heart failure using ECG signals. Appl. Intell..

[51] Wang L, Zhou W, Chang Q, Chen J, Zhou X (2019). Deep ensemble detection of congestive heart failure using short-term RR intervals. IEEE Access.

[52] Zhang Y, Yi J, Chen A, Cheng L (2023). Cardiac arrhythmia classification by time–frequency features inputted to the designed convolutional neural networks. Biomed. Signal Process. Control.

[53] Rahman A (2022). ECG classification for detecting ECG arrhythmia empowered with deep learning approaches. Comput. Intell. Neurosci..

[54] Grandini, M., Bagli, E., & Visani, G. Metrics for multi-class classification: an overview. *ArXiv*, Aug. 2020, Accessed: Nov. 16, 2023. [Online]. Available: https://www.semanticscholar.org/paper/Metrics-for-Multi-Class-Classification%3A-an-Overview-Grandini-Bagli/2c9022fe0af15568a885e59d475ec8f95726e51b.

